# Characterization of a Novel Orthomyxo-like Virus Causing Mass Die-Offs of Tilapia

**DOI:** 10.1128/mBio.00431-16

**Published:** 2016-04-05

**Authors:** Eran Bacharach, Nischay Mishra, Thomas Briese, Michael C. Zody, Japhette Esther Kembou Tsofack, Rachel Zamostiano, Asaf Berkowitz, James Ng, Adam Nitido, André Corvelo, Nora C. Toussaint, Sandra Cathrine Abel Nielsen, Mady Hornig, Jorge Del Pozo, Toby Bloom, Hugh Ferguson, Avi Eldar, W. Ian Lipkin

**Affiliations:** a Department of Cell Research and Immunology, The George S. Wise Faculty of Life Sciences, Tel Aviv University, Tel Aviv, Israel; b Center for Infection and Immunity, Mailman School of Public Health, Columbia University, New York, New York, USA; c New York Genome Center, New York, New York, USA; d Department of Poultry and Fish Diseases, The Kimron Veterinary Institute, Bet Dagan, Israel; e Easter Bush Pathology, The Royal (Dick) School of Veterinary Studies and The Roslin Institute, University of Edinburgh, Midlothian, Scotland; f Marine Medicine Program, Pathobiology, School of Veterinary Medicine, St. George's University, Grenada, West Indies

## Abstract

Tilapia are an important global food source due to their omnivorous diet, tolerance for high-density aquaculture, and relative disease resistance. Since 2009, tilapia aquaculture has been threatened by mass die-offs in farmed fish in Israel and Ecuador. Here we report evidence implicating a novel orthomyxo-like virus in these outbreaks. The tilapia lake virus (TiLV) has a 10-segment, negative-sense RNA genome. The largest segment, segment 1, contains an open reading frame with weak sequence homology to the influenza C virus PB1 subunit. The other nine segments showed no homology to other viruses but have conserved, complementary sequences at their 5′ and 3′ termini, consistent with the genome organization found in other orthomyxoviruses. *In situ* hybridization indicates TiLV replication and transcription at sites of pathology in the liver and central nervous system of tilapia with disease.

## IMPORTANCE

The economic impact of worldwide trade in tilapia is estimated at $7.5 billion U.S. dollars (USD) annually. The infectious agent implicated in mass tilapia die-offs in two continents poses a threat to the global tilapia industry, which not only provides inexpensive dietary protein but also is a major employer in the developing world. Here we report characterization of the causative agent as a novel orthomyxo-like virus, tilapia lake virus (TiLV). We also describe complete genomic and protein sequences that will facilitate TiLV detection and containment and enable vaccine development.

## INTRODUCTION

Tilapia are increasingly important to domestic and global food security. Comprising more than 100 species, Nile tilapia (*Oreochromis niloticus*) is the predominant cultured species worldwide (1). Global production is estimated at 4.5 million metric tons with a current value in excess of $7.5 billion U.S. dollars (USD) and is estimated to increase to 7.3 million metric tons by 2030 (2, 3). The largest producers (in order) are China, Egypt, Philippines, Thailand, Indonesia, Laos, Costa Rica, Ecuador, Colombia, and Honduras. The United States is the leading importer, consuming 225,000 metric tons of tilapia annually (4). In addition to their value as an inexpensive source of dietary protein (5, 6), tilapia have utility in alga and mosquito control and habitat maintenance for shrimp farming (7). A wide range of bacteria, fungi, protozoa, and viruses has been described as challenges to tilapiine aquaculture (8–10). Bacterial and fungal infections have been addressed through the use of antibiotics or topical treatments. No specific therapy has been described for viral infections of tilapia (11); however, viruses were not implicated as substantive threats until 2009, when massive losses of tilapia, presumed to be due to viral infection, were described in Israel and Ecuador (12, 13).

Eyngor and colleagues investigated outbreaks in Israel and reported a syndrome comprising lethargy, endophthalmitis, skin erosions, renal congestion, and encephalitis and demonstrated transmissibility of disease from affected to naive fish. They cultured a virus from infected fish in E-11 cells (cloned subculture of snakehead fish cell line), demonstrated sensitivity to ether and chloroform, and obtained a sequence specific for disease through cDNA library screening that predicted a 420-amino-acid (aa) open reading frame (ORF) with no apparent homology to any nucleic acid or protein sequence in existing databases. The virus was tentatively named tilapia lake virus lake virus (TiLV); however, no taxonomic assignment was feasible at that time (13). Ferguson and colleagues described a disease in farmed Nile tilapia in South America that differed from that caused by the Israeli virus in that pathology was focused in the liver rather than in the central nervous system (12). No agent was isolated; however, PCR using primers and probes based on the sequence obtained from the virus isolated in Israel revealed the presence of a similar virus (J. Del Pozo, N. Mishra, R. N. Kabuusu, S. Cheetham Brows, A. Eldar, E. Bacharach, W. I. Lipkin, H. W. Ferguson, unpublished data).

Here, we report comprehensive analysis of the TiLV isolate from Israel. Unbiased high-throughput sequencing (UHTS), Northern hybridization, mass spectrometry (MS), *in situ* hybridization, and infectivity studies indicate that TiLV is a segmented, negative-sense RNA virus. TiLV contains 10 genome segments, each with an open reading frame (ORF). Nine of the segments have no recognizable homology to other known sequences; one segment predicts a protein with weak homology to the PB1 subunit of influenza C virus, an orthomyxovirus. Our findings suggest that TiLV represents a novel orthomyxo-like virus and confirm that it poses a global threat to tilapiine aquaculture (12, 13).

## RESULTS

### High-throughput sequencing and bioinformatic analysis.

RNA extracts of brain from tilapia with disease in Israel were depleted of rRNA and were used as the template for Ion Torrent sequencing. RNA extracted from nuclease-treated, sucrose gradient-purified and concentrated particles from infected E-11 culture cells were used as the template for Illumina sequencing. Reads from two Ion Torrent and two Illumina libraries were taxonomically classified using taxMaps (https://github.com/nygenome/taxmaps) by mapping against the National Center for Biotechnology Information’s (NCBI) nucleotide database, the NCBI RefSeq database (14), the tilapia reference genome sequence (Orenil1.1), and corresponding annotated tilapiine mRNA sequences (15). Unclassified reads (not mapping to any known sequence) were then independently assembled using the VICUNA assembler (16). Contigs from each library were aligned with BLAST (17) against all contigs from the other 3 libraries, retaining hits with an E value of 1e−10 or lower to identify assembled sequences likely to derive from the same segment of the same species of virus in different samples. Single-linkage clustering was used to group together all of the contigs that showed any similarity. We identified 10 contig clusters that contained at least one contig in each of the 4 libraries. Within each cluster, contigs were aligned to each other and manually assembled to generate a maximum-length sequence after inverted tandem duplications at the ends of contigs, likely resulting from amplification artifacts, were removed. Overlapping predicted open reading frames (ORFs) in contigs from different assemblies were used to correct for frameshift errors and to infer the longest possible ORF. Based on a model wherein the genomic segments are anticipated to contain conserved termini, we used a combination of k-mer analysis, read depth analysis, and manual curation to build 5′- and 3′-terminal sequence motifs to refine terminal sequences. Mapping of the initial raw read data against the 10 final consensus sequences with BWA-MEM (17) demonstrated that 99% of the unidentified reads from the Illumina libraries and 87% of unidentified reads from the Ion Torrent libraries mapped to the consensus sequences.

### Characterization of TiLV genome.

**FIG 1:**
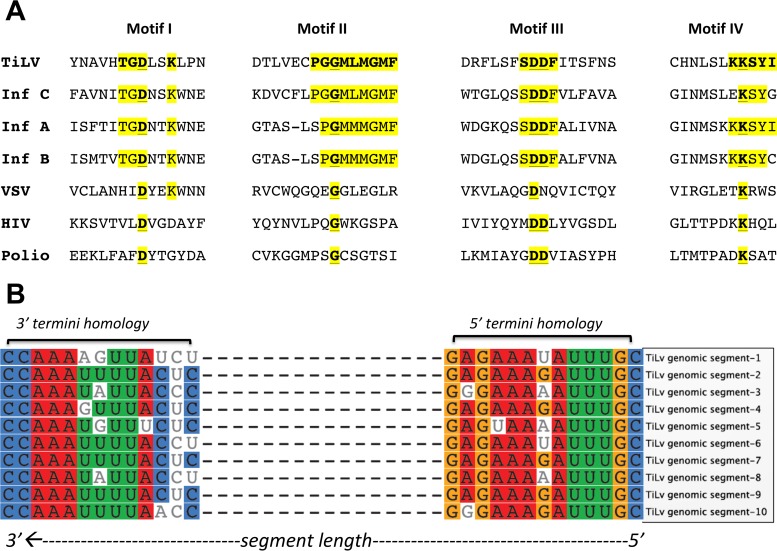
Genomic characterization of the segments of TiLV isolated from tilapia in Israel. (A) TiLV segment 1 putative protein shows weak homology to motifs conserved in RNA-dependent polymerases. Sequence comparison of TiLV’s segment 1 predicted protein with motifs I to IV, conserved in polymerases of influenza virus strains C/JJ/50 (Inf C) (19), A/WSN/33 (Inf A) (34), and B/Lee/40 (Inf B) (35), vesicular stomatitis virus (VSV) (20), human immunodeficiency virus (HIV) (21), and poliovirus (Polio) (22). The relative motif positions are also shown. Invariant sequences in each motif are in boldface and underlined. TiLV sequences that show identity to one of the influenza virus sequences are highlighted in yellow. (B) Genomic segments of TiLV show conserved and homologous features at 5′ and 3′ termini.

PCR primers were designed and used to amplify fragments representing all 10 contigs from RNA extracts from infected fish and purified virus particles. 5′ and 3′ rapid amplification of cDNA ends (RACE) was used to recover terminal sequences in all 10 clusters. Based on similarity in terminal sequences in the individual clusters, we concluded that the clusters represented 10 viral genomic segments and henceforth will refer to them as segments 1 to 10 (GenBank accession no. KU751814 to KU751823) (Table 1). Segment 1 is the largest at 1.641 kb. Segments 2 to 10 are 1.471, 1.371, 1.250, 1.099, 1.044, 0.777, 0.657, 0.548, and 0.465 kb, respectively. Segment 1 predicted a protein with weak homology to the PB1 subunit of influenza C virus (~17% amino acid identity, 37% segment coverage) (see Fig. S1 in the supplemental material). The other nine segments had no homology to other sequences in the GenBank nucleotide and protein databases with mega blast/BLASTx/tBLASTn or tBLASTx. Based on weak homology with PB1 motifs (18–23), we compared the segment 1 ORF with polymerase subunit PB1 recovered from specific members of the *Orthomyxoviridae* family. Segment 1 putative protein showed low homology to four motifs conserved in RNA-dependent RNA and RNA-dependent DNA polymerases (Fig. 1A).

**TABLE 1:** Genomic characterization of 10 segments of TiLV isolated from tilapia in Israel

Segment no.	Segment length (nt)	GenBank accession no.	Predicted protein length (aa)	Protein mol mass (kDa)	pI	Identification by MS^a^
1	1,641	KU751814	519	57.107	7.99	−
2	1,471	KU751815	457	51.227	9.64	+
3	1,371	KU751816	419	47.708	7.99	+
4	1,250	KU751817	356	38.625	9.01	+
5	1,099	KU751818	343	38.058	8.46	+
6	1,044	KU751819	317	36.381	8.67	+
7	777	KU751820	195	21.834	9.98	+
8	657	KU751821	174	19.474	9.86	+
9	548	KU751822	118	13.486	6.49	+
10	465	KU751823	113	12.732	4.45	+

a Table S1 in the supplemental material describes peptides identified by mass spectrometry.

The nucleotide sequences of the 5′ and 3′ noncoding regions of all 10 segments were aligned using Geneious software 6.8.1 (Fig. 1B). Thirteen nucleotides at the 5′ termini and 13 nucleotides at the 3′ termini were similar in all segments, an organization similar to that observed for influenza viruses (24, 25). Ten of 13 nucleotides on the 5′ termini and 7 of 13 at the 3′ termini had 100% conservation; the remaining nucleotides were conserved in segments 5 to 9. Nucleotide sequences showed complementary features at the 5′ and 3′ termini. Predicted molecular and biochemical features for individual viral segments are provided in Table 1. Mass spectrometry of virus isolated from E-11 cells confirmed the ORFs and coding peptides predicted for segments 2 to 10. No protein sequence was detected that represented segment 1 (Table 1; see Table S1 in the supplemental material). ORFs predicted from segment 5 and segment 6 contained signal peptide cleavage sites between aa 25/26 and 30/31, respectively.

High-throughput sequencing and alignment analyses of a TiLV PCR-positive sample from a fish in Ecuador showed 97.20 to 99.00% nucleotide identity and 98.70 to 100% amino acid identity to the corresponding coding region of nucleotide sequences obtained with samples from Israel (Table 2).

**TABLE 2:** Comparison of nucleotide and amino acid similarities of TiLV in Ecuador and Israel

Segment no.	GenBank accession no. (Israel TiLV)	Predicted ORF length (aa)	Total no. of nt mutations in coding region^a^^,^^b^	% identity with TiLV in coding region		aa change(s) in TiLV from Israel vs TiLV from Ecuador^a^
				nt	aa	
1	KU751814	519	44 (37 s, 7 ns)	97.20	98.70	K41→R, V85→I, G98→S, R104→K, V130→I, K207→R, P515→L
2	KU751815	457	31 (33 s, 4 ns)	97.70	99.10	G4→E, I61→T, I228→V, R236→K
3	KU751816	419	22 (22 s, 0 ns)	98.40	100.00	None
4	KU751817	356	25 (21 s, 4 ns)	97.80	98.90	V33→A, A35→V, S275→L, N319→D
5	KU751818	343	15 (12 s, 3 ns)	98.50	99.10	S4→A, I17→T, R111→K
6	KU751819	317	17 (12 s, 3 ns)	98.20	99.10	Y8→C, S27→N, I227→V
7	KU751820	195	12 (11 s, 1 ns)	98.00	100	F135→L
8	KU751821	174	7 (6 s, 1 ns)	98.70	99.40	G42→S
9	KU751822	118	4 (3 s, 1 ns)	99	99	D109→N
10	KU751823	113	4 (3 s, 1 ns)	99	99	G52→S

a Submitted GenBank sequences of TiLV isolated from Israel.

b s, synonymous mutation; ns, nonsynonymous mutation.

### Northern hybridization experiments indicate that TiLV is a segmented RNA virus.

**FIG 2:**
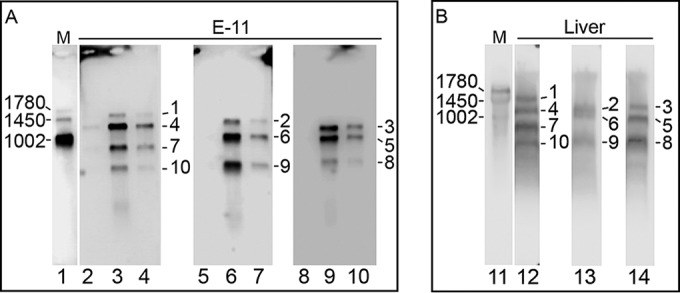
Northern hybridization analysis indicates that TiLV is a segmented RNA virus. (A) Total RNA extracted from E-11 cells 6 days postinfection with TiLV from brains of tilapia in Israel (lanes 3, 6, and 9), from virions that were pelleted from the culture supernatant (lanes 4, 7, and 10), or from noninfected E-11 cells (lanes 2, 5, and 8). (B) Total RNA extracted from livers of tilapia in Ecuador (lanes 12 to 14). The extracts were hybridized to probe mixtures representing segments 1, 4, 7, and 10 (probe mixture 1 [lanes 2 to 4 and 12]), segments 2, 6, and 9 (probe mixture 2 [lanes 5 to 7 and 13]), or segments 3, 5, and 8 (probe mixture 3 [lanes 8 to 10 and 14]) to prevent signal overlap from segments of similar sizes. Influenza A virus RNA (A/Moscow/10/99) hybridized with three probes representing hemagglutinin (HA) (1,780 nt), NA (1450 nt), and matrix (1,002 nt) sequences served as size references (M [lanes 1 and 11]). Size markers appear on the left sides of the panels and segment numbers on the right.

The segmented nature of TiLV and the sizes of specific segments were confirmed in Northern hybridization analyses of extracts from E-11 cells infected with TiLV (brain isolate) (Fig. 2A) and from liver of infected fish (Fig. 2B), using 10 segment-specific, discrete probes.

### *In situ* hybridization.

**FIG 3:**
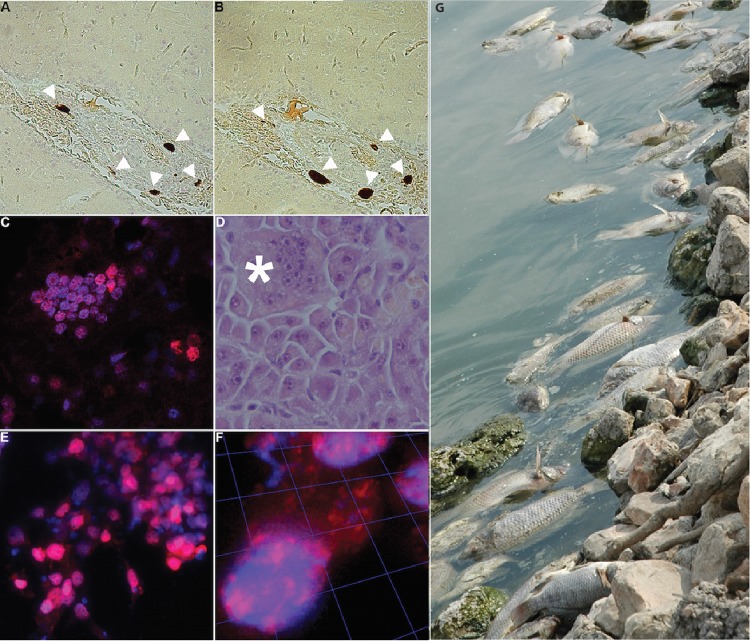
Detection of TiLV RNA in brain and liver of infected tilapia and infected E-11 cells by *in situ* hybridization and image of dead tilapia in Israel. (A and B) Brain sections of infected Nile tilapia hybridized with Affymetrix Cy3-conjugated probes (red) of various polarities to TiLV segment 1 to detect genomic RNA (A) or mRNA (B). White arrowheads indicate hybridization signal. (C) Liver sections hybridized with Cy3-conjugated (red) Stellaris probes to segment 3 to detect mRNA. Nuclei are stained with DAPI (blue). (D) Liver section stained with hematoxylin and eosin reveals multinucleated giant cells (asterisk). (E) TiLV-infected E-11 cells hybridized with Quasar 670-conjugated (red) Stellaris probe to segment 3 to detect TiLV mRNA. Nuclei are stained with DAPI (blue). (F) Images of confocal sections of cells in panel E were reconstituted into a 3D image. (G) Dead tilapia at a fish farm in Israel.

To investigate the presence of TiLV RNA in diseased fish, we applied *in situ* hybridization techniques to both liver and brain samples with TiLV-specific probes. In the brain, hybridization signals for segment 1 genomic RNA (Fig. 3A, arrowheads) and mRNA (Fig. 3B, arrowheads) were confined to the leptomeninges, mostly adjacent to blood vessels. Similar results were obtained for genomic RNA and mRNA of segment 5 (data not shown). No hybridization signal was detected in sections of brains of uninfected healthy controls (naive fish) of approximately the same age collected from a different breeding facility in Israel (see Fig. S2A in the supplemental material). In the liver, hybridization signal for segment 3 mRNA was detected in hepatocytes. Many nuclei were clustered, suggesting the formation of multinucleated cells (Fig. 3C and D). To image infected cells with high resolution, E-11 cultures were infected with TiLV, hybridized with segment 3-derived probes to detect viral mRNA, and imaged. TiLV mRNA was detected in both the nucleus and cytoplasm of multiple cells (Fig. 3E and F). No viral mRNA was detected in noninfected control cells (see Fig. S2B).

### TiLV has a negative-sense genome.

**FIG 4:**
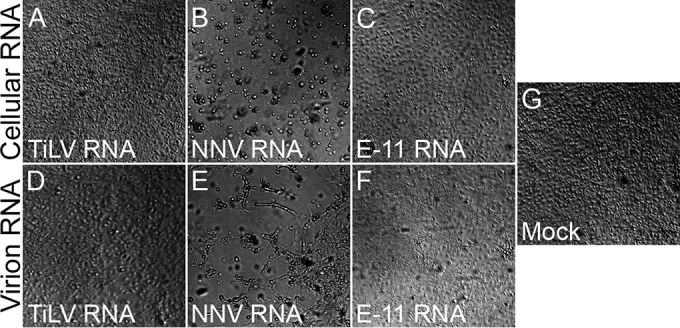
TiLV deproteinized RNA is not infectious. Naive E-11 cell cultures were transfected with deproteinized RNA, extracted from cultured cells (“Cellular RNA” [A to C]) or pellets of culture supernatants (“Virion RNA” [D to F]), from TiLV-infected (“TiLV RNA” [A and D]) or NNV-infected (“NNV RNA” [B and E]) E-11 cells, or from naive E-11 cells (“E-11 RNA” [C and F]). Transfection with no RNA (“Mock” [G]) was also included. Bright-field images of transfected cultures were taken at 8 days posttransfection.

Based on sequence and RNA hybridization analysis, we postulated that TiLV has a negative-sense genome. Accordingly, a deproteinized genome should not be infectious. RNA was purified from TiLV virion pellets or from TiLV-infected E-11 cells and was transfected into naive E-11 cells. For positive controls, naive E-11 cells were transfected with nervous necrosis virus (NNV [a positive-sense, segmented RNA virus of fish]) RNA extracts from NNV-infected cells or from NNV virions. No cytopathic effect (CPE) was observed following transfection of deproteinized TiLV RNA extracted from cells or virions (Fig. 4A and D), similar to the lack of cytopathic effect upon transfection of control RNA extracted from naive E-11 cell culture (Fig. 4C), from pellets of the culture supernatant (Fig. 4F), or in mock-transfected cells (Fig. 4G). In contrast, transfection of deproteinized NNV RNA, extracted from infected cells (Fig. 4B) or from virions (Fig. 4E), resulted in cytopathic effect in E-11 cells.

## DISCUSSION

This study was undertaken to characterize the molecular biology, pathogenesis, and partial geographic distribution of TiLV, a novel virus recently implicated in large die-offs of tilapia in Israel and Ecuador (Fig. 3G) (12, 13). Efforts to classify TiLV through classical sequence homology analyses of sequences obtained from infected fish and cultured cells had failed; thus, we pursued Northern hybridization, mass spectrometry, and *in situ* hybridization to identify and correlate the presence of candidate viral genes and proteins. Results presented here indicate that TiLV is an RNA virus with a genome comprised of 10 unique segments. The largest segment has minimal homology to the PB1 segment of the orthomyxovirus influenza virus C. It also contains the major polymerase motifs (19). We speculate therefore that segment 1 encodes the polymerase of TiLV. The other 9 segments have no apparent homology to other known viral sequences; however, the presence of complementary sequences at their termini and identification of proteins in extracts of infected cells that correlate with the ORFs they carry provide evidence that they represent gene segments of TiLV. Sequence analyses provide no insight into their functions in the TiLV life cycle. *In situ* hybridization experiments indicated a nuclear site for transcription.

It is likely that TiLV will ultimately be classified as representing a new genus of the family *Orthomyxoviridae*. As noted above, the 5′ and 3′ noncoding termini of TiLV include 13 nucleotides that are similar in all segments. This organization resembles that observed for the influenza, Thogoto, and infectious salmon anemia orthomyxoviruses (24–27) and enables base pairing and formation of secondary structures important for replication, transcription, and packaging of viral RNA. In addition, all of the 5′ ends of TiLV genomic RNA segments contain a short, uninterrupted uridine stretch (3 to 5 bases long). This feature is reminiscent of the stretch of 5 to 7 uridine residues found in other orthomyxoviruses, on which the viral polymerase “stutters” while generating poly(A) tails (28, 29).

The presence of viral nucleic acid at sites of pathology in brain and liver together with the observation that virus propagated in cell culture is capable of inducing disease in naive fish implicates TiLV as the causative agent of outbreaks of both viral encephalitis and syncytial hepatitis in tilapiines in Israel and Ecuador. The fact that TiLV has been detected in association with disease in two geographically disparate sites underscores that TiLV poses a global threat to tilapiine aquaculture. We have no evidence that TiLV can infect other species; however, the genetic dissimilarity of TiLV to other viruses suggests the potential for discovery of related viruses as TiLV sequences are employed for phylogenetic analysis.

## MATERIALS AND METHODS

### Nucleic acid extraction, library preparation, and high-throughput sequencing.

Unbiased high-throughput sequencing was performed on Illumina HiSeq 2500 and PGM Ion Torrent platforms. For library preparations, total RNA was extracted from purified virus particles and infected fish brain tissue samples with TRI reagent (Sigma-Aldrich, St. Louis, MO). RNA extractions from TiLV-infected tissues included postextraction DNase I (2 U/µg DNA for 15 min at 37^0^C; Thermo, Fisher, Waltham, MA) and depletion of rRNA sequences with RiboZero magnetic kits (Illumina, San Diego, CA). High-throughput sequencing was performed in parallel on random-primed cDNA preparations from purified virus and infected brain tissue RNA. Sequencing from purified virus on the Illumina HiSeq 2500 platform (Illumina) resulted in an average of ~200 million reads (100 nucleotides [nt]) per sample. Total RNA was reverse transcribed using SuperScript III (Thermo, Fisher) with random hexamers. The cDNA was RNase H treated prior to second-strand synthesis with Klenow fragment (New England Biolabs, Ipswich, MA). The resulting double-stranded cDNA mix was sheared to an average fragment size of 200 bp using the manufacturer’s standard settings (E210 focused ultrasonicator; Covaris, Woburn, MA). Sheared product was purified (AxyPrep Mag PCR cleanup beads; Axygen/Corning, Corning, NY), and libraries were constructed using KAPA library preparation kits (KAPA, Wilmington, MA) with 6-nt bar code adapters. The quality and quantity of libraries were checked using Bioanalyzer (Agilent, Santa Clara, CA). Samples were demultiplexed using Illumina-supplied CASAVA software and exported as FastQ files. More than 90% of Illumina reads passed the Q30 filter. Demultiplexed FastQ files were assembled to generate reads, contigs, and clusters. Sequencing on the Ion Torrent PGM platform was performed with Ion PGM Sequencing 200 kits on Ion 318 chips (Life Technologies, Carlsbad, CA), yielding on average ~2 million reads per sample, with a mean length of 177 nt. For Ion Torrent, cDNA preparations were sheared (Ion Shear Plus kit; Life Technologies) for an average fragment size of 200 bp and added to Agencourt AMPure XP beads (Beckman Coulter, Brea, CA) for purification, libraries were prepared with KAPA library preparation/Ion Torrent series kits (KAPA), and emulsion PCR was performed with Ion PGM Template OT2 200 kits (Life Technologies). Ion Torrent reads were demultiplexed and exported as FastQ files by the Ion Torrent PGM software. After bar code and adaptor trimming, length filtering, masking of low-complexity regions, and subtraction of ribosomal and host sequences, reads were mapped as described for Illumina data.

### Mass spectrometry.

TiLV virions were purified by ultracentrifugation through 25% (wt/vol) sucrose cushions. The samples were trypsinized and analyzed by liquid chromatography-tandem mass spectrometry (LC-MS/MS) on Q Exactive Plus (Thermo Scientific). Peptides were identified by Discoverer software version 1.4, using the Sequest search engine, the Uniprot database, and the putative TiLV proteins as references. All of these analyses were performed at the Smoler Proteomics Center, Technion, Israel.

### Northern hybridization analysis.

Fragments representing each segment were amplified by PCR using primers shown in Table S2 in the supplemental material. Amplified PCR products were purified with QIAquick gel extraction kit (Qiagen, Hilden, Germany) and labeled with biotin with the Deca Label DNA labeling kit (Thermo, Fisher Scientific) (30). RNA was extracted from E-11 cells infected with TiLV from brains of tilapia from Israel and from livers of infected tilapia from Ecuador. RNA extracts were size fractionated by electrophoresis on a denaturing 1.5% agarose gel, transferred to Biobond-Plus nylon transfer membrane (Sigma-Aldrich), and UV cross-linked. Membranes were hybridized overnight in a low-shaker incubator with biotinylated probe combos (1 or 2 and 3) at 65°C in 6× SSC (1× SSC is 0.15 M NaCl plus 0.015 M sodium citrate) (31, 32). Biotin-labeled probes were detected with the chemiluminescent nucleic acid detection module kit (Thermo, Fisher Scientific). Blots were scanned with a C DiGit chemiluminescence blot scanner (LI COR).

### *In situ* hybridization.

To detect TiLV RNA, *in situ* hybridizations were performed with ViewRNA probes (Affymetrix, Santa Clara, CA) and/or Stellaris fluorescent probes (Biosearch Technologies, Novato, CA) in tissue sections and cultured infected or noninfected cells. Tissue samples were collected from euthanized infected or uninfected fish and fixed in 10% neutral buffered formalin. Specimens were embedded in paraffin and serially sectioned (5 µm thick), fixed in 10% formaldehyde (Fisher Scientific), deparaffinized, boiled in pretreatment solution (Affymetrix), and digested with proteinase K (Affymetrix). Sections were hybridized for 3 h at 40°C with custom-designed QuantiGene ViewRNA probes (Affymetrix). Bound probes were then amplified per protocol from Affymetrix using PreAmp and Amp molecules. Multiple-label probe oligonucleotides conjugated to alkaline phosphatase (LP-AP type 1) were then added, and Fast Red substrate was used to produce signal (red dots, Cy3 fluorescence). Infected and noninfected cultured cells, grown on 2% gelatin-coated glass coverslips, were fixed with 3.7% formaldehyde. Negative-sense probes, derived from segment 3, labeled with Cy3 or with Quasar 670 fluorophores, and compatible with the Stellaris system, were constructed to detect TiLV mRNA (see Table S3 in the supplemental material). When applied, nuclei were counterstained with DAPI (4′,6-diamidino-2-phenylindole). Sections were examined by light microscopy (Axio Scope.A1; Carl Zeiss), fluorescence microscopy (Nikon TE200 or Zeiss Axiovert 200 M), and confocal microscopy with three-dimensional (3D) image reconstruction (33).

### Infectivity of deproteinized RNA.

Confluent E-11 cell cultures in 25-cm^2^ flasks were infected, or not, with TiLV or NNV (one flask per virus). Cells and culture supernatants were collected upon the appearance of cytopathic effect (CPE) (2 or 3 days postinfection for NNV or TiLV, respectively). Deproteinized RNA was prepared from cells using EZ-RNA reagent (Biological Industries, Israel), according to the manufacturer’s instructions. Supernatants were cleared by centrifugation (200 × *g* at room temperature for 5 min), and virions were pelleted by ultracentrifugation (107,000 × *g* at 4°C for 2 h) through a 25% (wt/vol) sucrose cushion. Each pellet was resuspended in 140 µl phosphate-buffered saline (PBS), and deproteinized RNA was extracted using QIAamp viral RNA isolation kit (Qiagen). E-11 cells (~80% confluence in 6-well plates) were transfected with the 2.5 µg of cellular deproteinized RNA or with the entire deproteinized virion RNA preparation, using the TransIT-mRNA transfection kit (Mirus Bio LLC, Madison, WI).

### Sequence accession numbers.

TiLV sequences are available at GenBank under the following accession numbers: KU751814, KU751815, KU751816, KU751817, KU751818, KU751819, KU751820, KU751821, KU751822, KU751823.

## SUPPLEMENTAL MATERIAL

Figure S1  Amino acid alignment. TiLV segment 1 putative protein amino acid alignment with influenza C polymerase subunit PB1 shows partial and low homology (~17% identity, ~37% coverage). Download Figure S1, PDF file, 0.1 MB

Figure S2  (A) Brain section from naive Nile tilapia was processed as described in the legend to Fig. 3A. (B) Uninfected E-11 cells were processed as described in the legend to Fig. 3E. Download Figure S2, TIF file, 2.7 MB

Table S1  TiLV coding peptides identified by mass spectrometry. Table S1, XLSX file, 0.01 MB

Table S2  Primers used to generate probes for Northern hybridization experiments. Table S2, XLSX file, 0.1 MB

Table S3  Sequences of probes to segments 1, 3, and 5 used for *in situ* hybridization experiments. Table S3, XLSX file, 0.01 MB
