# Supplementary material for: Characterization of a Novel Orthomyxo-like Virus Causing Mass Die-Offs of Tilapia
Source: mBio. 2016 Apr 5;7(2):e00431-16. doi: 10.1128/mBio.00431-16 (PMC4959514; doi:10.1128/mBio.00431-16)
Supplement: Figure S1 — Amino acid alignment. TiLV segment 1 putative protein amino acid alignment with influenza C polymerase subunit PB1 shows partial and low homology (~17% identity, ~37% coverage). Download [file mbo002162777sf1.pdf]

Figure S1.

```
TiLV-Segment-1-RDRP        1 -----MWAFQEG-----VCKGNLLSGPTSMKAPDSA-------------ARESLDRASEI  37
                                     F        +      +GP  M    S              +R S+++ S++
Influenza c virus-PB1      1 MEINPYLMFLNNDVTSLISTTYPYTGPPPMSHGSSTKYTLETIKRTYDYSRTSVEKTSKV  60

TiLV-Segment-1-RDRP       38 MT----------------GKSYNAVHTGDLSKLPN-----QGESPLRIVDSDLYSE----  72
                                                K    V  G L  L        GE   +    +  +E
Influenza c virus-PB1     61 FNIPRRKFCNCLEDKNELVKPTGNVDIGSLLGLAEMMEKRMGEGFFKHCVMEAETEILKM 120

TiLV-Segment-1-RDRP       73 ---------RSCCWVIEKEG------RVVCKSTTLTRG-MTGLLNTTRCSSPSELI---- 112
                                      ++  W  E+        ++   +   T G   G      C+   E++
Influenza c virus-PB1    121 HFSRLTEGRQTYDWTSERNMPAATALQLTVDAIKETEGPFKGTTMLEYCNKMIEMLDWKE 180

TiLV-Segment-1-RDRP      113 CKVLTVESLSEK------------------IGDTSVEELLSHGRYFKCALRDQERGKPKS 154
                              K   V+++  +                  I     +E L          +D ERGK +
Influenza c virus-PB1    181 IKFRKVKTMVRREKDKRSGKEIKTKVPVMGIDSIKHDEFLIRALTINTMAKDGERGKLQR 240

TiLV-Segment-1-RDRP      155 RAIFLSHPFFRLLSSVVETHARSVLSKVSAVYTATASAEQRAMMAAQVVESRKHV----- 209
                             RAI       R  S +VET A+ +  K+          E++A +   V      +
Influenza c virus-PB1    241 RAIATPGMIVRPFSKIVETVAQKICEKLKESGLPVGGNEKKAKLKTTVTSLNARMNSDQF 300

TiLV-Segment-1-RDRP      210 ---LNGDCTKYNEAIDADTLLKVWDAIGMGSI-----------------------GVMLA 243
                                + GD +K+NE    +  L +   I   S                        G+ L+
Influenza c virus-PB1    301 AVNITGDNSKWNECQQPEAYLALLAYITKDSSDLMKDLCSVAPVLFCNKFVKLGQGIRLS 360

TiLV-Segment-1-RDRP      244 YMVRRKCVLIK---------------DTLVE----------C--PGGMLMGMFNATAT-- 274
                                + K V+IK                 L E          C  PGGMLMGMFN  +T
Influenza c virus-PB1    361 NKRKTKEVIIKAEKMGKYKNLMREEYKNLFEPLEKYIQKDVCFLPGGMLMGMFNMLSTVL 420

TiLV-Segment-1-RDRP      275 -----------LALQGTTDRFLSFSDDFITSFNSPAELREIEDLL---FASCH----NLS 316
                                        L  +G     L  SDDF+  F   +    I   +    A C     N+S
Influenza c virus-PB1    421 GVSTLCYMDEELKAKGCFWTGLQSSDDFVL-FAVASNWSNIHWTIRRFNAVCKLIGINMS 479

TiLV-Segment-1-RDRP      317 LKKSYISVASLEINSCTLTRDGDLATGL----------GCTAGVPFRGPLVTLKQTAAML 366
                             L+KSY S+  L     ++  DG+  + L          G   GV F   +  +K
Influenza c virus-PB1    480 LEKSYGSLPEL-FEFTSMFFDGEFVSNLAMELPAFTTAGVNEGVDFTAAMSIIKTNMINN 538

TiLV-Segment-1-RDRP      367 SGAVDSGVMPFHSAERLFQIKQQECAYR----------YNNPTYTTRNEDFLPTCLGGKT 416
                             S +  + +M      + F+   +   +            N    T  N+D L    GGK
Influenza c virus-PB1    539 SLSPSTALMALRICLQEFRATYRVHPWDSRVKGGRMKIINEFIKTIENKDGLLIADGGKL 598

TiLV-Segment-1-RDRP      417 VISFQSL------LTWD-------------CHPF--------WYQVHPDGPDTIDQK--V 447
                             + +  +L      L ++              +PF         ++ H  GP  +++   V
Influenza c virus-PB1    599 MNNISTLHIPEEVLKFEKMDEQYRNRVFNPKNPFTNFDKTIDIFRAH--GPIRVEENEAV 656

TiLV-Segment-1-RDRP      448 LSVLASKTRRRRTRLEA------------------------LSDLDPLVPHRLLVSESDV 483
                             +S  + +TR  RT L                           +D++P  P   +     +
Influenza c virus-PB1    657 VSTHSFRTRANRTLLNTDMRAMMAEEKRYQMVCDIFKSVFESADINP--PIGAMSIGEAI 714

TiLV-Segment-1-RDRP      484 SKIRAARQAHLKSLGLEQPTNFNYAIYKAVQPTAGC----- 519
                              +    R    + +G  + + +   I   ++
Influenza c virus-PB1    715 EEKLLERAKMKRDIGAIEDSEYE-EIKDIIRDAKKARLESR 754
```
