# Supplementary figures and images for: Characterization of a Novel Orthomyxo-like Virus Causing Mass Die-Offs of Tilapia
Source: mBio. 2016 Apr 5;7(2):e00431-16. doi: 10.1128/mBio.00431-16 (PMC4959514; doi:10.1128/mBio.00431-16)

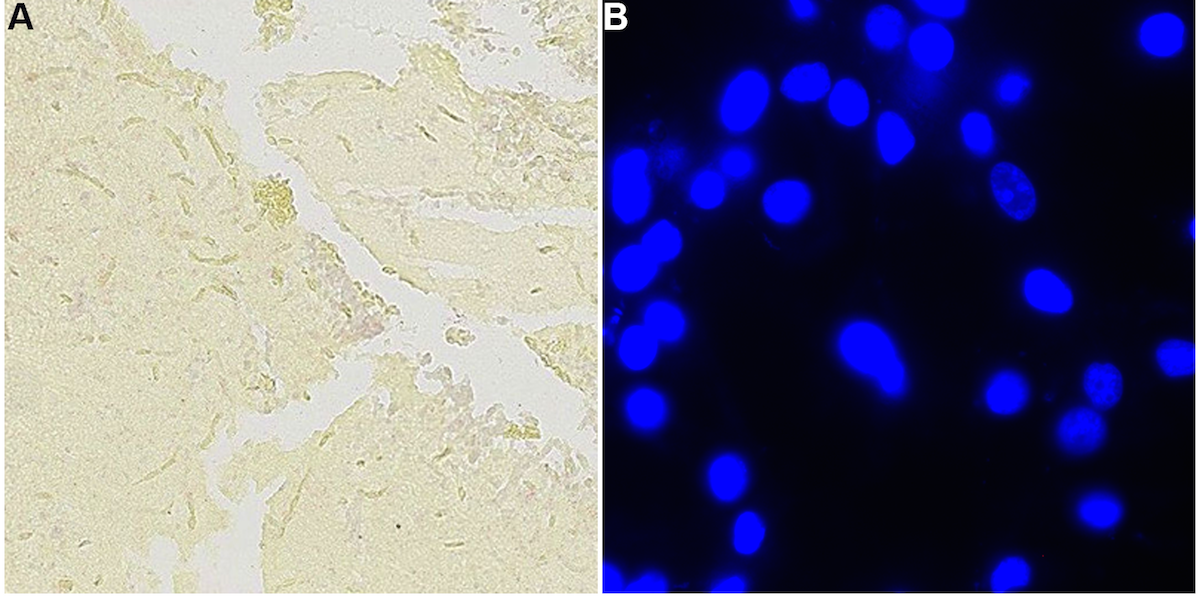

Supplement: Figure S2 — (A) Brain section from naive Nile tilapia was processed as described in the legend to Fig. 3A. (B) Uninfected E-11 cells were processed as described in the legend to Fig. 3E. Download [file mbo002162777sf2.tif]
